# The *Drosophila* chromosomal protein Mst77F is processed to generate an essential component of mature sperm chromatin

**DOI:** 10.1098/rsob.160207

**Published:** 2016-11-03

**Authors:** Shuhei Kimura, Benjamin Loppin

**Affiliations:** Laboratoire de Biométrie et Biologie Evolutive, CNRS UMR5558, University of Lyon, Université Claude Bernard Lyon 1, Villeurbanne, France

**Keywords:** Mst77F, protamine, spermiogenesis, genome compaction, protein processing, convergent evolution

## Abstract

In most animals, the bulk of sperm DNA is packaged with sperm nuclear basic proteins (SNBPs), a diverse group of highly basic chromosomal proteins notably comprising mammalian protamines. The replacement of histones with SNBPs during spermiogenesis allows sperm DNA to reach an extreme level of compaction, but little is known about how SNBPs actually function *in vivo*. Mst77F is a *Drosophila* SNBP with unique DNA condensation properties *in vitro*, but its role during spermiogenesis remains unclear. Here, we show that Mst77F is required for the compaction of sperm DNA and the production of mature sperm, through its cooperation with protamine-like proteins Mst35Ba/b. We demonstrate that Mst77F is incorporated in spermatid chromatin as a precursor protein, which is subsequently processed through the proteolysis of its N-terminus. The cleavage of Mst77F is very similar to the processing of protamine P2 during human spermiogenesis and notably leaves the cysteine residues in the mature protein intact, suggesting that they participate in the formation of disulfide cross-links. Despite the rapid evolution of SNBPs, sperm chromatin condensation thus involves remarkably convergent mechanisms in distantly related animals.

## 1. Introduction

Spermiogenesis, the differentiation of post-meiotic spermatids into mature spermatozoa, generally involves major cellular reorganization events, such as the elimination of cytoplasm and the growth of a sperm flagellum [1]. Differentiating spermatids also undergo dramatic changes at the nuclear level, with the progressive acquisition of species-specific nuclear shape, which is often associated with extreme reduction of the nuclear volume. At the molecular level, sperm DNA compaction is achieved through the replacement of nucleosomes with sperm nuclear basic proteins (SNBPs), such as the well-known mammalian protamines [2–4]. *Drosophila* is an excellent model for the study of sperm chromatin remodelling at the functional level, as the process shares several key features with the mammalian histone-to-protamine transition [5]. First, in *Drosophila*, as in most mammals, the vast majority of nucleosomes are replaced by SNBPs, with the notable exception of epigenetic determinants of sperm centromere identity [6–9]. Second, histones are transiently replaced with transition proteins before the final deposition of SNBPs [7,10]. Third, fly SNBPs and protamines of eutherian mammals are enriched in cysteine residues, which, in mammals, are known to form stabilizing intermolecular disulfide bonds [3,4,11]. Finally, the mechanism of de novo assembly of paternal nucleosomes at fertilization is remarkably conserved and specifically involves the HIRA histone chaperone complex [12–16].

Protamine 1 (P1) is a small SNBP of about 50 AA, which is found in all mammals. In some species, such as human and mouse, sperm chromatin additionally contains protamines of the Protamine 2 (P2) family [17]. In contrast with P1, protamine P2 is expressed as a precursor, which is subsequently processed by proteolysis to generate P2, P3 and P4, differing only by their N-terminal extension of a few residues [18,19]. P1 and P2 are encoded by single genes and are essential for sperm DNA integrity and male fertility in mouse [20,21]. *Drosophila* sperm chromatin seems to harbour a larger diversity of SNBPs. In *D. melanogaster*, three types of SNBPs have been so far identified: Male-specific-transcript-35Ba/b (Mst35Ba/b) (also known as ProtA/B), Male-specific transcript 77F (Mst77F) and Protamine-like 99C (Prtl99C) [11,22]. In contrast with arginine-rich mammalian protamines, *Drosophila* protamine-like SNBPs are equally enriched in arginine and lysine residues. Interestingly, fly SNBPs are also characterized by the presence of a truncated high-mobility group (HMG) box motif, which is apparently specific to this group of proteins [23].

In mouse, invalidation of one copy of *protamine 1* or *protamine 2* is sufficient to induce male sterility [20]. By sharp contrast, the complete absence of both *Mst35Ba/b* paralogues does not prevent male fertility [24,25], thus indicating that other *Drosophila* SNBPs can at least partially compensate for the lack of Mst35Ba/b. Indeed, it was recently reported that Mst35Ba/b functionally cooperate with Prtl99C for sperm DNA compaction. Interestingly, Prtl99C is itself essential for mature sperm production and male fertility, thus revealing the existence of a functional hierarchy between fly SNBPs [22].

The third group of SNBPs present in mature sperm chromatin is represented by its founding member, Mst77F, and its paralogues encoded by several gene copies present on the Y-chromosome, and collectively referred to as *Mst77Y* genes [11,26–28]. The *Mst77F* gene encodes a relatively large SNBP of 215 residues, which is incorporated into spermatid nuclei at the histone-to-protamine transition. The original functional characterization of *Mst77F* concluded that this gene is required for male fertility [11]. In addition, it was proposed that Mst77F controls the proper shaping of spermatid nuclei [11,24]. A legitimate concern with these conclusions is that they were essentially based on the analysis of a single point mutant allele of *Mst77F* that apparently behaved as an antimorph [11,24]. Furthermore, a recent study provided evidence that Mst77F had the ability to efficiently aggregate DNA *in vitro*, suggesting that the protein could play a role in sperm nuclear compaction rather than nuclear shaping [29].

In this work, we reinvestigated the function of *Mst77F* using newly generated loss-of-function alleles. We demonstrate that Mst77F is required for the proper compaction of spermatid chromatin following the histone-to-protamine transition. Finally, we show that Mst77F is proteolytically processed during spermiogenesis, in a way remarkably similar to mammalian P2.

## 2. Results

### 2.1. Generation of null *Mst77F* alleles using CRISPR/Cas9 gene targeting

The original functional characterization of *Mst77F* was based on the analysis of two mutant alleles: a hypomorphic *PiggyBac* insertion in the *Mst77F* promoter (*Mst77F^c06969^*) and a point mutation (*Mst77F^1^*) that causes the S149T amino acid substitution in Mst77F protein [11]. *Mst77F^1^* was actually identified on a chromosome bearing the unmapped *ms(3)nc3* male sterile mutation [30]. Using a deficiency of the region, *Df(3L)ri-79c,* it was reported that *Mst77F^1^/Df(3L)ri-79c* males were sterile and fail to produce mature sperm. Furthermore, spermatids in these males arrested their differentiation after the histone-to-protamine transition and displayed an aberrant ellipsoid shape [11,24]. However, the combinatorial analysis of these three genetic elements led to the conclusion that *Mst77F^1^* behaved as an antimorphic allele, thus raising doubts about the actual function of Mst77F during spermiogenesis.

**Figure 1.**
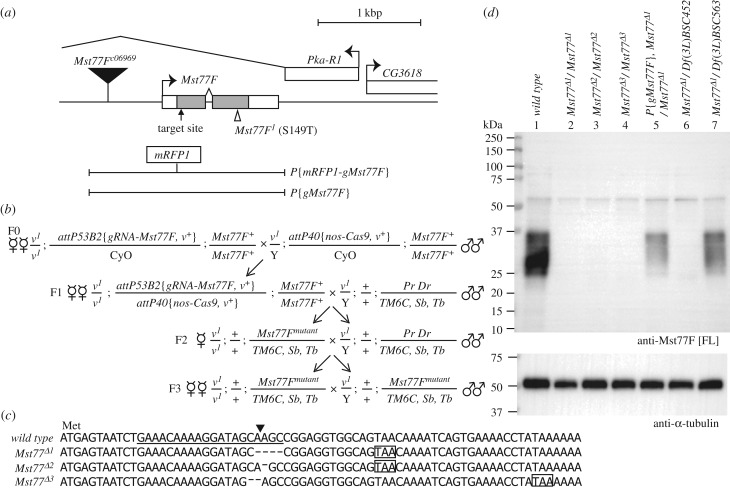
*Mst77F* mutant generation by CRISPR/Cas9. (*a*) Scheme of the genomic region of *Mst77F*. *Mst77F* is located within a large intron of the *Pka-R1* gene. The respective positions of the *Mst77F^c06969^* Piggybac insertion (black triangle) and a point mutation (S149T) in *Mst77F^1^* (white triangle) are indicated. Two rescue transgenes (*P{gMst77F}* and *P{mRFP1-gMst77F}*) are shown. In *P{mRFP1-gMst77F}*, the *mRFP1* coding sequence is inserted upstream the *Mst77F* start. The 20-bp target site of CRISPR/Cas9 is localized after the *Mst77F* start (arrow). (*b*) Crossing scheme for generating small deletion alleles in *Mst77F* using the CRISPR/Cas9 system. (*c*) Alignment sequences of three individual *Mst77F* mutants. Each of the small deletion induces the formation of a premature stop codon (rectangles). The CRISPR/Cas9 target sequence is underlined and the cutting site indicated (black triangle). (*d*) Western blotting analysis of testicular extracts of the indicated genotypes using the anti-Mst77F [FL] antibody. The Mst77F protein is detected as a smear (25 – 37 kDa), which could reflect the existence of post-translational modifications. Anti-α-tubulin antibody is used as loading control.

We thus turned to clustered regularly interspaced short palindromic repeats/CRISPR associated protein 9 (CRISPR/Cas9) gene targeting system [31] to obtain new *Mst77F* mutant alleles. We generated flies expressing a single guide RNA (gRNA) that targets a 20 bp sequence starting 11 bp downstream from the translational start codon of *Mst77F* (figure 1*a*) in order to create frameshift mutations after the initiation codon. These flies were crossed with a stock expressing Cas9 protein specifically in the germline [32]. The targeting of *Mst77F* was only performed in females to avoid any undesired off-target effect on the closely related *Mst77Y* genes present on the Y chromosome (figure 1*b*). Among the 15 putative *Mst77F* mutant alleles obtained (see Material and methods), three were randomly selected and sequenced. All three showed a small deletion around the target site, which created a frameshift and a premature stop codon (figure 1*c*). Western blotting (WB) analysis using a polyclonal antibody raised against the full-length protein (anti-Mst77F [FL]) confirmed that Mst77F was undetectable in testicular extracts of homozygous mutant males (figure 1*d*). We concluded that these new alleles, named *Mst77F^Δ1^*, *Mst77^Δ2^* and *Mst77F^Δ3^*, are null or at least strong loss-of-function alleles. All three alleles induce complete male sterility at the homozygous state. We noted, however, that trans-heterozygous combinations of these mutant alleles occasionally produced rare progeny (about 0.5% of the control) (table 1). Although the origin of this difference is unclear, we speculate that it could reflect putative off-target effects of the CRISPR/Cas9 endonuclease complex. Note, however, that fertility of homozygous *Mst77F^Δ1^* males was restored with a genomic *Mst77F* transgene (*P{gMst77F}*) (figure 1*a* and table 1), thus indicating that the *Mst77F^Δ1^* chromosome does not carry any other male sterile mutation.

**Table 1.** Fertility tests.

genotype	average no. of adult progeny
*+/+*	204.0
*Mst77F^Δ1^/TM6C*	158.8
*Mst77F^Δ1^/Mst77F^Δ1^*	0
*Mst77F^Δ2^/Mst77F^Δ2^*	0
*Mst77F^Δ3^/Mst77F^Δ3^*	0
*P{gMst77F}, Mst77F^Δ1^/Mst77F^Δ1^*	65.2
*Mst77F^Δ1^/Mst77F^Δ2^*	0.8
*Mst77F^Δ1^/Mst77F^Δ3^*	0.8
*Mst77F^Δ2^/Mst77F^Δ3^*	0.6
*Mst77F^Δ1^/Df(3L)BSC452*	1.5
*Mst77F^Δ1^/Df(3L)BSC563*	122.8
*P{gMst77F}, Mst77F^Δ1^/Df(3L)BSC452*	81.7
*Mst77F^Δ1^/Df(3L)ri-79c*	126.2
*Mst77F^Δ1^/Mst77F^c06969^*	104.6
*P{gMst77F}, Mst77F^Δ1^/Mst77F^Δ2^*	131.3
*P{gMst77F-EGFP}/+; Mst77F^Δ1^/Mst77F^Δ2^*	0
*P{mRFP1-gMst77F}/+; Mst77F^Δ1^/Mst77F^Δ2^*	40.2
*ΔMst35B/CyO*	113.1
*ΔMst35B/ΔMst35B*	78.6
*ΔMst35B/CyO; Mst77F^Δ1^/TM2*	91.9
*ΔMst35B/ΔMst35B; Mst77F^Δ1^/TM2*	0.1
*ΔMst35B/ΔMst35B; Mst77F^Δ1^/+, P{gMst77F}*	111.8

As expected, *Df(3L)BSC452*, a deficiency that uncovers *Mst77F* (see FlyBase, http://flybase.org), did not complement *Mst77F* mutant alleles. Surprisingly, however, *Df(3L)ri-79c* (the deficiency used for the original characterization of the *Mst77F* phenotype [11]) fully complemented *Mst77F^Δ1^* sterility (table 1). Consistent with this result, genomic DNA sequencing and WB analyses unambiguously showed that *Df(3L)ri-79c* does not uncover *Mst77F* and does not affect its normal expression (electronic supplementary material, figures S1*a* and *b*). Similarly, we showed that the *Mst77F^c06969^* Piggybac insertion complemented *Mst77F^Δ1^* sterility (table 1) and did not prevent the expression of Mst77F (electronic supplementary material, figure S1*b*). Unfortunately, we have not been able to obtain the *Mst77F^1^* allele, which is probably no longer available.

We conclude that the previously reported sterility of *Mst77F^1^/Df*(*3L*)*ri-79c* males is probably unrelated to Mst77F function. Analysis of the new *Mst77F^Δ^* alleles however establishes that Mst77F is importantly required for male fertility.

### 2.2. *Mst77F* mutant spermatids fail to complete spermiogenesis

**Figure 2.**
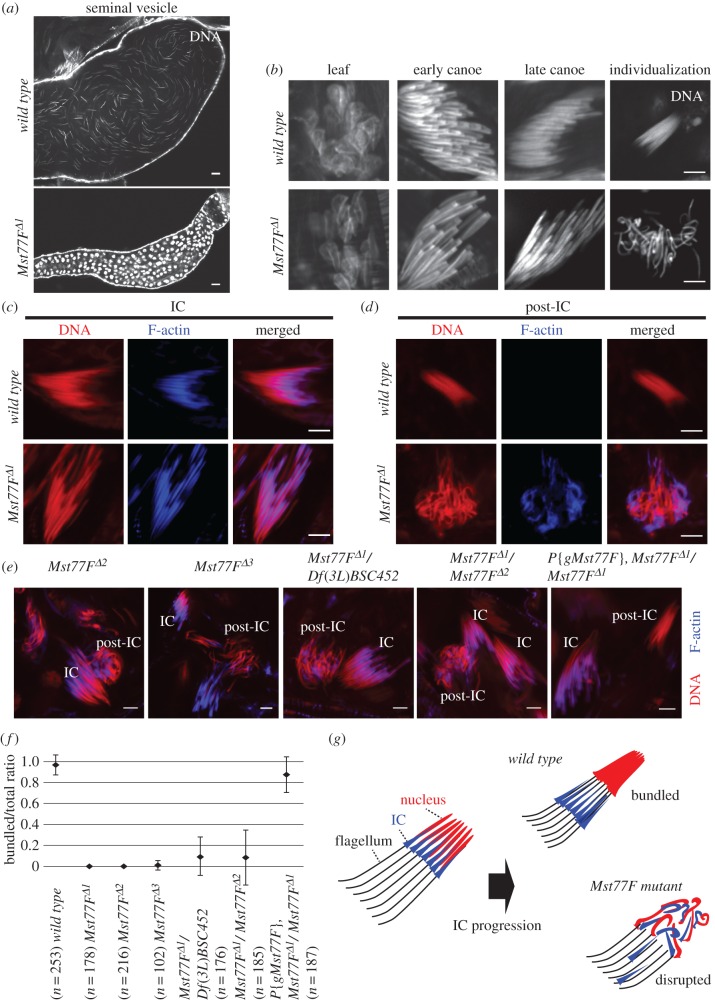
Spermatid nuclear bundles are disrupted after IC appearance in *Mst77F* mutant. (*a*–*e*) Confocal images of whole-mount testes and seminal vesicles. (*a*) Seminal vesicle. In wild-type adult males, seminal vesicles are full of mature sperm, but are empty in *Mst77F^Δ1^* mutant. Scale bars, 10 µm. (*b*) Spermatid nuclei at leaf, early canoe, late canoe and individualization stage stained for DNA. Scale bars, 5 µm. (*c*) Spermatid nuclei at the individualization complex (IC) stage stained for DNA (red) and fluorescently labelled phalloidin (blue). (*d*) Spermatid nuclei during or after IC progression (post-IC) stained as in (*c*). In wild-type testes, after the passage of the IC, spermatid nuclei appear tightly bundled. By contrast, in *Mst77F^Δ1^* mutant, IC remains associated with aberrantly shaped nuclei. (*e*) IC and post-IC stages in *Mst77F^Δ2^*, *Mst77F^Δ3^*, *Mst77F^Δ1^/Df(3L)BSC452*, *Mst77F^Δ1^/Mst77F^Δ2^* and *Mst77F^Δ1^* mutant rescued by *P{gMst77F}* stained as in (*c*). Scale bars in (*c*–*e*) 5 µm. (*f*) Quantification of the morphology of post-IC spermatid nuclei. Averages of normal/total post-IC spermatid nuclear bundles ratio are shown. Error bars indicate standard deviation (SD). (*g*) Schematic representation of *Mst77F* mutant phenotype.

The observation of spermatogenesis in adult *Mst77F* mutant males revealed the absence of mature sperm in seminal vesicles, thus explaining the sterility phenotype (figure 2*a*). As expected, the initiation of spermiogenesis (the differentiation of post-meiotic spermatids) was not affected by the absence of Mst77F. Groups of 64 elongating spermatid nuclei were indistinguishable in wild-type and *Mst77F* mutant males until the late canoe stage, shortly before individualization (figure 2*b*). Following this stage, bundles of mutant spermatids appeared severely disorganized compared with the tightly clustered control spermatids (figure 2*b*). We then stained testes with fluorescently labelled phalloidin to reveal the actin-based individualization complexes (IC) (figure 2*c*). Individualization occurs with the progression of these structures from nuclei toward the distal tip of flagella [33–36]. In wild-type testes, groups of spermatid nuclei remained tightly clustered during and after the passage of the IC (upper panels in figures 2*c*,*d*). In *Mst77F* mutant testes, however, individualization was systematically associated with the progressive disorganization of spermatid bundles and abnormal spermatid nuclei morphology. Indeed, many nuclei appeared bent and improperly compacted (figure 2*d*, lower panel). Identical defects were observed in all tested *Mst77F* allelic combinations and the phenotype was fully rescued by the *P{gMst77F}* transgene (figure 2*e*). Note, however, that a fraction of apparently normal post-IC spermatid bundles were observed in trans-heterozygous mutant testes (figure 2*f*; electronic supplementary material, table S1). These observations correlate with the fact that the corresponding males occasionally produce rare progeny (table 1). We conclude that loss of Mst77F generally affects the morphology of spermatid nuclei, which probably prevents normal progression of individualization and sperm maturation (figure 2*g*).

### 2.3. Defective chromatin organization in *Mst77F* spermatids after the histone-to-protamine transition

**Figure 3.**
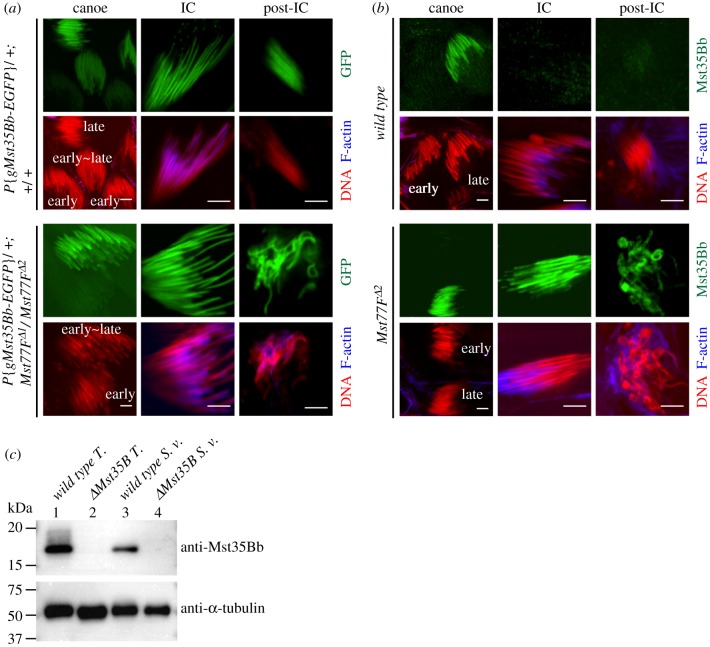
Absence of Mst77F does not prevent Mst35Bb incorporation but results in less compacted sperm chromatin. (*a*,*b*) Confocal images of spermatid nuclei of the indicated genotypes. DNA (red). F-actin (blue). Scale bars, 5 µm. (*a*) Mst35Bb-EGFP fluorescence (green) is detected in spermatid nuclei at late canoe stage, and persists after IC and post-IC stages in wild-type and mutant testes. (*b*) Anti-Mst35Bb (green) staining is never detected after IC stage in wild-type testes, whereas the antibody stains *Mst77F* mutant spermatid nuclei at their terminal stage. (*c*) Anti-Mst35B western blotting analysis of testes or seminal vesicles of the indicated genotypes. Anti-α-tubulin antibody is used as loading control. T., testes; S. v., seminal vesicles.

The nuclear defects of *Mst77F* mutant spermatids were first detected after the late canoe stage, which normally corresponds to the histone-to-protamine transition. To determine the consequence of Mst77F loss on this process, we stained control and mutant testes with histone and SNBP markers. We observed that histones were properly removed between early and late canoe stages in *Mst77F* mutants (electronic supplementary material, figure S2*a*). Similarly, incorporation and removal of the transition-like protein Tpl94D [7,10], a *Drosophila* equivalent of mammalian transition proteins, appeared unaffected by the absence of Mst77F (electronic supplementary material, figure S2*b*). We then analysed the distribution of the almost identical Mst35Ba and Mst35Bb SNBPs (collectively named Mst35Ba/b), using a transgene expressing Mst35Bb-EGFP. In wild-type testes, the Mst35Bb-EGFP fluorescence was first detected in the nuclei of late-canoe-stage spermatids and the nuclear signal remained very bright throughout spermiogenesis, as previously reported [11] (figure 3*a*, upper panel). Interestingly, Mst35Bb-EGFP was properly incorporated in *Mst77F* mutant spermatids and remained present even after the occurrence of nuclear defects (figure 3*a*, lower panel). These results indicate that the histone-to-protamine transition occurs independently of Mst77F.

We additionally performed immunostainings using an anti-Mst35Bb polyclonal antibody directed against the full-length protein [25] (see Material and methods). In wild-type testes, this antibody stained spermatid nuclei around the late canoe stage, as expected, but not at later stages (figure 3*b*, upper panel). The absence of staining after the IC stage probably reflects the inaccessibility of antibodies to highly condensed spermatid and sperm nuclei [37]. Indeed, WB experiments confirmed that Mst35Bb was present in protein extracts from seminal vesicles, which only contain mature sperm (figure 3*c*). Surprisingly, in *Mst77F* mutants, we noted that anti-Mst35Bb staining persisted on nuclear bundles that were associated with ICs and was even detected in late, aberrantly shaped nuclei (figure 3*b*, lower panel). Although we cannot exclude that the absence of Mst77F simply unmasks Mst35Bb antigens, these observations alternatively suggest that Mst77F mutant spermatid nuclei fail to condense properly after the histone-to-protamine transition, thus allowing accessibility of antibodies to spermatid chromatin.

It has been recently proposed that Mst77F is required for the incorporation of Mst35Ba and Mst35Bb in spermatid chromatin [23]. The origin of this discrepancy with our own results is unclear. It may lie in the fact that Doyen *et al*. used RNAi to knock-down *Mst77F* expression, which could have additionally impacted the expression of highly related *Mst77Y* genes. In any case, our analysis of *Mst77F* loss of function alleles clearly demonstrates that the incorporation of Mst35Ba/b occurs independently of Mst77F.

In their study, Doyen *et al*. [23] additionally proposed that Mst77F incorporation into spermatid chromatin is mediated by a testis-specific analogue of the histone chaperone Nap1, *tNap1* (also named *hanabi /CG5017*). However, their report of the *tNAP1* RNAi KD phenotype differed substantially from the previously reported phenotype of *hanabi* null allele [38]. Notably, in *hanabi^1^* mutants, the loss of spermatid clustering occurs earlier and spermatid nuclei are found scattered all over the elongating cyst. In addition, although we confirmed that histones were correctly removed in *hanabi^1^* spermatids (electronic supplementary material, figure S3*c*), two independent anti-Mst77F antibodies clearly detected nuclear incorporation of Mst77F in this mutant (electronic supplementary material, figure S3*a*,*a*′). The same result was obtained using the Mst77F-EGFP transgene (electronic supplementary material, figure S3*a*″). Our results thus indicate that Mst77F deposition in spermatid chromatin occurs independently of *tNAP1/Hanabi*.

### 2.4. Mst77F and Mst35B cooperate for the organization of sperm chromatin

Our analysis of *Mst77F* loss-of-function alleles establishes that this SNBP is required for male fertility. By clear contrast, it has been previously shown that the absence of Mst35Ba/b proteins does not prevent the formation of functional male gametes [24,25], suggesting that these proteins are at least partially redundant with another SNBP for the packaging of sperm DNA. We thus searched for a genetic interaction between our *Mst77F* alleles and *ΔMst35B*, a small deletion that precisely removes both *Mst35Ba/b* paralogues [25].

**Figure 4.**
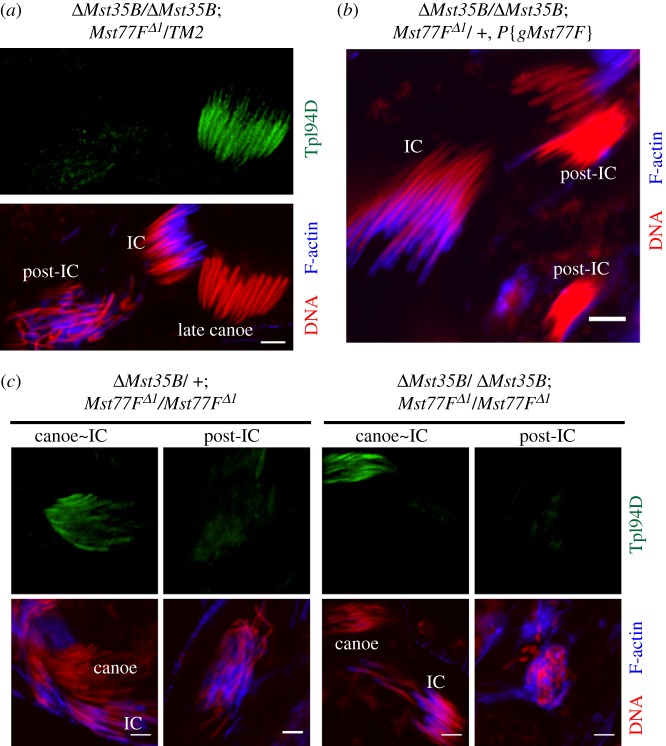
*Mst35Ba/b* and *Mst77F* genetic interaction. (*a*–*c*) Confocal images of spermatid nuclei. DNA (red). F-actin (blue). Anti-Tpl94D (green). (*a*) *ΔMst35B/ ΔMst35B; Mst77F^Δ1^/+* mutant showing aberrantly organized nuclei at the post-IC stage. Tpl94D incorporation and removal appears normal. (*b*) *P{gMst77F}* rescues the normal progression of the IC complex on spermatid nuclei in *ΔMst35B/ΔMst35B; Mst77F^Δ1^/+* mutant testes. (*c*) Tpl94D removal from late canoe spermatids occurs independently of Mst35Ba/b and Mst77F. Scale bars, 5 µm.

We first observed that *ΔMst35B/+; Mst77F^Δ1^*/+ double heterozygous males are fertile (table 1). Remarkably, however, the elimination of one *Mst77F* allele in a *ΔMst35B* homozygous background almost completely abolished male fertility and was associated with spermiogenesis defects (table 1 and figure 4*a*). The phenotype of spermatid nuclei in these animals was indistinguishable from the spermatid nuclear defects observed in single *Mst77F* homozygous mutants. Importantly, a single copy of *P{gMst77F}* rescued the male sterility induced by this interaction (table 1 and figure 4*b*). This genetic interaction strongly suggests that Mst77F and Mst35Ba/b cooperate for the proper compaction of spermatid nuclei following the histone-to-protamine transition.

Finally, we observed that Tpl94D was normally removed from spermatid nuclei after the canoe stage in *ΔMst35B/ ΔMst35B; Mst77F^Δ1^/+* (figure 4*a*). Furthermore, the removal of one or both copies of the *Mst35Ba/b* locus did not aggravate the spermiogenesis defects of *Mst77F* homozygous mutant males, and Tpl94D was normally removed from spermatid nuclei after the canoe stage (figure 4*c*).

### 2.5. Mst77F is processed by proteolysis during spermiogenesis

**Figure 5.**
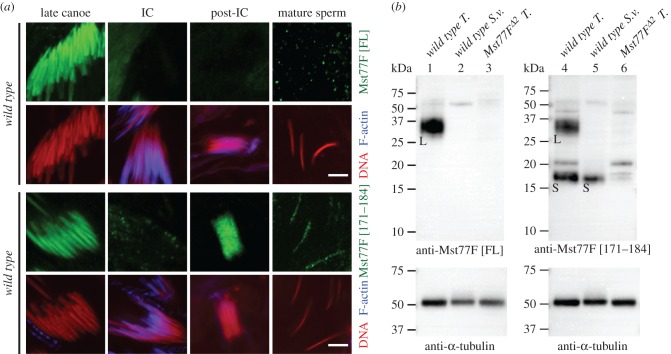
Mst77F is proteolytically processed during spermiogenesis. (*a*) Confocal images of spermatid nuclei in testes and mature sperm nuclei in seminal vesicles. DNA (red). F-actin (blue). The anti-Mst77F [FL] antibody (green) detects Mst77F at the late canoe stage but not later (upper panels). The anti-Mst77F [171–184] (green) antibody detects Mst77F throughout late spermiogenesis with the exception of the IC stage (lower panels). Scale bars, 5 µm, (*b*) Western blotting analyses of wild-type testes or seminal vesicles using the indicated anti-Mst77F antibodies. Anti-α-tubulin antibody is used as loading control. T., testes; S. v., seminal vesicles. Specific bands are labelled as follows: L, smear bands (between 25 kDa and 37 kDa) corresponding to the larger form of Mst77F protein; S, a band (approximately 17 kDa) corresponding to the smaller form of Mst77F protein.

The distribution of Mst77F in testes was originally established using a transgene expressing an Mst77F-EGFP fusion protein that localized in spermatid nuclei from late canoe stage onwards. In addition, Mst77F-EGFP was transiently detected in flagella from the canoe stage until individualization [11]. Immunostainings with the anti-Mst77F [FL] antibody recapitulated both the nuclear and flagellar distribution of Mst77F-EGFP, with the exception of late spermatid and sperm nuclei (figures 5*a*, upper panel; electronic supplementary material, figure S4A, upper panel). Interestingly, although this staining was mostly absent in *Mst77F* mutant testes, a faint residual signal was nevertheless observed in late-canoe-stage spermatid nuclei (electronic supplementary material, figure S4*a*, middle and lower panels). Although Mst77F is encoded by a unique autosomal gene, the *D. melanogaster* Y chromosome harbours at least 18 highly related *Mst77Y* genes, including several potentially functional copies [27,28]. It is thus possible that the anti-Mst77F [FL] antibody recognizes a putative Mst77Y protein, which would explain the residual staining observed in *Mst77F* mutant testes. Note however that the antibody did not detect any putative Mst77Y protein in WB (figure 1*d*).

We also used an anti-Mst77F [171–184] antibody that was raised against a peptide from the C-terminus of Mst77F protein [24]. Interestingly, in contrast with anti-Mst77F [FL], anti-Mst77F [171–184] antibody decorates Mst77F on spermatid nuclei even after IC formation and the staining persists in mature sperm, as previously shown (figures 5*a*, lower panel; electronic supplementary material, figure S4B, upper panel) [24]. In protein extracts from dissected testes (without seminal vesicles), both antibodies detected the same Mst77F band, which migrated between 25 and 37 kDa (Mst77F predicted size is 24.5 kDa) (figure 5*b*, lane 1 and 4). Surprisingly, the anti-Mst77F [171–184] antibody additionally detected an abundant, specific band of approximately 17 kDa (figure 5*b*, lane 4). Moreover, only this smaller Mst77F band was detected in extracts prepared from seminal vesicles, which only contain mature sperm (figure 5*b*, lane 5). Taken together, these results suggest that Mst77F is synthesized as a precursor that undergoes proteolytic maturation, with only the shorter form persisting in mature sperm.

**Figure 6.**
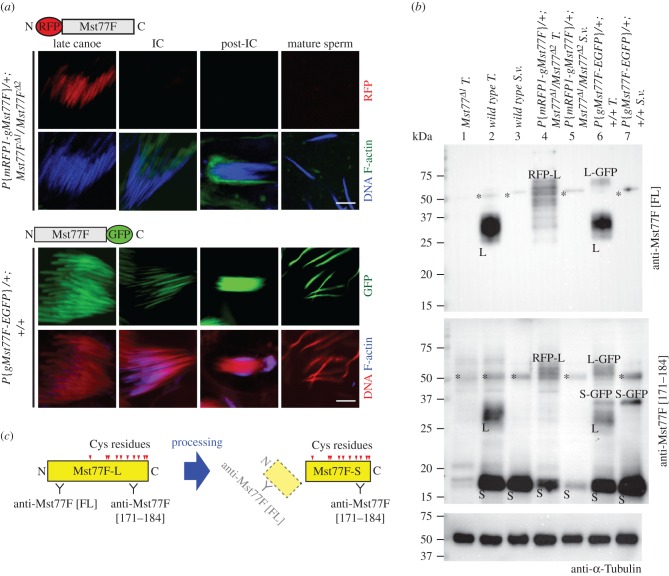
Mst77F is processed from its N-terminus after its deposition in spermatid chromatin. (*a*) Confocal images of spermatid nuclei in testes and mature sperm nuclei in seminal vesicles. Upper panels: mRFP1-Mst77F fluorescence (red) in *Mst77F* mutant background disappears after the late canoe stage. DNA (blue). F-actin (green). Lower panels: Mst77F-EGFP is detected throughout late spermiogenesis and in mature sperm nuclei. DNA (red). F-actin (blue). Scale bars: 5 µm. (*b*) Western blotting analyses using the Mst77F [FL] or Mst77F [171–184] antibodies. Anti-α-tubulin antibody is used as loading control. L, larger form of Mst77F protein; S, smaller form of Mst77F protein; RFP-L, large mRFP1-Mst77F; L-GFP, large Mst77F-GFP; S-GFP, truncated Mst77F-EGFP protein. Asterisks indicate non-specific bands. T., testes; S. v., seminal vesicles. (*c*) A model for the processing of Mst77F. The precursor Mst77F protein (Mst77F-L) is processed just before or during individualization. All 10 cysteine residues of Mst77F (red triangles) are retained in the mature form of Mst77F (Mst77F-S), which packages sperm chromatin.

The fact that the Mst77F-EGFP protein (with the EGFP fused to the C-terminus of Mst77F) remains present in mature sperm nuclei in a wild-type background (figure 6*a*, lower panel) is in apparent support of the processing of Mst77F from its N-terminus. However, the *Mst77F-EGFP* transgene failed to rescue the fertility and the spermiogenesis defects of *Mst77F* mutant males, despite the apparent incorporation of this recombinant protein in spermatid nuclei (table 1; electronic supplementary material, figure S5). The presence of EGFP thus seems to interfere with the normal function of mature Mst77F. We thus constructed a new transgene that expresses Mst77F tagged in its N-terminus with monomeric red fluorescent protein (*P{mRFP1-gMst77F}*) (figure 1*a*). In contrast with *Mst77F-EGFP*, the *mRFP1-Mst77F* transgene rescued the fertility of *Mst77F* mutant males (table 1). We then analysed the distribution of mRFP1-Mst77F during spermiogenesis in rescued males by detecting the mRFP1 fluorescence. We first observed the expected incorporation of mRFP1-Mst77F in late canoe stage spermatid nuclei (figure 6*a*, upper panel). Strikingly however, the mRFP1-Mst77F nuclear fluorescence completely disappeared at the onset of individualization (figure 6*a*, upper panel), thus recapitulating the immunostainings obtained with the anti-Mst77F [FL] antibody. To confirm that N-terminal processing of the recombinant protein induced the loss of mRFP1 fluorescence, we analysed protein extracts from testes and seminal vesicles of rescued males in WB. In testicular extracts stained with the anti-Mst77F [171–184] antibody, the 25–37 kDa Mst77F band (L) shifted to an apparent size of about 55 kDa (figure 6*b*, middle panel, lane 4, RFP-L), as expected from the presence of the mRFP1 tag (expected size of the recombinant protein: 60 – 65 kDa). However, the size of the small band (S) remained unchanged (figure 6*b*, middle panel, lane 4). In seminal vesicles of the same males, the full-length recombinant protein was not detected but the short isoform was still detected (figure 6*b*, middle panel, lane 5). Finally, we also analysed protein extracts from testes and seminal vesicles of *Mst77F-EGFP* males (in a *Mst77F* wild-type background). Interestingly, a specific band migrating at an intermediary position (about 37 kDa) was detected with anti-Mst77F [171–184] antibody in both testes and seminal vesicles of *Mst77F-EGFP* males (figure 6*b*, middle panel, lane 6 and 7, S-GFP), thus indicating that the recombinant protein is processed like the endogenous Mst77F. By contrast, the S-GFP band was not detected using the anti-Mst77F [FL] antibody (figure 6*b*, upper panel, lane 6 and 7). Thus, both immunofluorescence and western blot analyses indicate that the anti-Mst77F [FL] antibody recognizes an epitope in the N-terminus and can thus be considered specific to pre-Mst77F (figure 6*c*).

Our results thus demonstrate that Mst77F is first synthetized as a precursor protein (pre-Mst77F), which is processed by proteolysis of its N-terminus to generate the mature form of Mst77F present in sperm chromatin.

## 3. Discussion

The analysis of loss-of-function alleles of *Mst77F* demonstrates that this SNBP is required for male fertility by allowing the proper organization of sperm chromatin following the histone-to-protamine transition. We observed that, in the absence of Mst77F, spermatid nuclei appeared normal up until the passage of the IC. The observed disruption of *Mst77F* mutant spermatid nuclei during individualization suggests that incomplete nuclear compaction physically disturbs the passage of IC around nuclei. However, we cannot exclude the possibility that, incomplete DNA compaction indirectly triggers the arrest of spermiogenesis through a putative checkpoint of spermatid chromatin state.

Our phenotypic analysis established that Mst77F functionally cooperates with Mst35Ba/b for sperm chromatin compaction. What makes Mst77F important for spermiogenesis remains, however, an open question, especially when considering that Mst77Y copies are probably expressed and translated in the male germline (note that *Mst35Y* copies are also present on the Y chromosome, but they so far appear non-functional [39]). At least, our genetic analysis rules out the possibility that Mst77Y proteins efficiently compensate for the loss of Mst77F.

**Figure 7.**
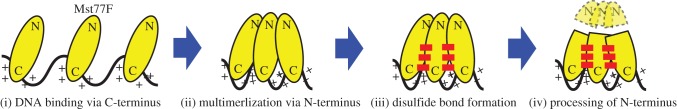
Model of sperm chromatin compaction and stabilization by Mst77F. (i) Mst77F (yellow ovals) first binds DNA through its C-terminus as proposed by Kost *et al*. [29]. (ii) DNA-bound Mst77F proteins multimerize through N-terminus interactions [29]. (iii) Mst77F intermolecular interactions are stabilized by disulfide bonds (red rectangles) connecting cysteine residues. (iv) Mst77F N-termini are finally removed by proteolysis, leaving only Mst77F-S on sperm chromatin.

In the course of this work, we have also discovered that Mst77F is proteolytically processed through a yet unknown mechanism. Despite the lack of homology between Mst77F and mammalian protamines, this mechanism is remarkably similar to the processing of protamine P2 in human and mouse. In these species, P2 is indeed processed from its N-terminus following its incorporation into chromatin [18,19,40–42]. Although the exact length of mature Mst77F is not known, proteolysis of the N-terminus probably removes about 30% of the precursor protein, based on our WB analyses. If this estimation is correct, it implies that all 10 cysteine residues of Mst77F are retained in the processed protein (figure 6*c*). The clustering of cysteine residues in the C-terminus region of Mst77F is again remarkably similar to mouse and human P2 that retain all their cysteines after the maturation process [18,41]. This is a strong indication that these residues are indeed important for the function of mature Mst77F, presumably through their ability to form disulfide bonds. *In vitro*, Mst77F was shown to interact with DNA with its C-terminal domain, which triggered in turn the multimerization of the protein via the N-terminus domain, eventually forming protein–DNA aggregates [29]. If this model holds true *in vivo*, we speculate that the putative N-terminal multimerization domain could be eliminated by proteolysis after Mst77F deposition and its subsequent stabilization with intermolecular disulfide bonds (figure 7).

In summary, our results demonstrate that Mst77F is importantly required for sperm DNA compaction and sperm maturation in *D. melanogaster*. The convergent acquisition of highly similar N-terminal processing for Mst77F and P2 suggests that this mechanism of SNBP maturation plays a general and important role for the functional organization of sperm DNA. In fact, perturbation of P2 processing has been correlated with some forms of human infertility [19]. Future work should aim at understanding the molecular basis and function of SNBP maturation.

## 4. Material and methods

### 4.1. Fly stocks

*w^1118^* stock was used as a wild-type strain. *Mst77F-EGFP*, *Mst35Bb-EGFP* stocks were provided by Renate Renkawitz-Pohl. *ΔMst35B* and *hanabi^1^* were previously described [25,38]. The fly stocks for the Cas9 system (NIG-Fly #CAS-0001, #TBX-0007 and #TBX-0010) were obtained from National Institute of Genetics Fly Stock Center. *Df(3L)BSC563* (#25721) [43], *Df(3L)BSC452* (#24956) [43], *Df(3L)ri-79c* (#3127), *Mst77F^c06969^* (#17792) were obtained from the Bloomington Drosophila Stock Center. Flies were maintained on standard medium at 25°C.

### 4.2. Mutant generation

Mst77F mutants were generated as previously described [32]. The 20 bp target sequence (GAAACAAAAGGATAGCAAGC) in *Mst77F* was selected using Cas9 Target Finder (see http://www.shigen.nig.ac.jp/fly/nigfly/cas9/cas9TargetFinder.jsp). A pair of the primers (5′-CTTCGAAACAAAAGGATAGCAAGC-3′ and 5′-AAACGCTTGCTATCCT TTTGTTTC-3′) were annealed and inserted into the *pBFv-U6.2* vector digested in the *BbsI* sites. Then, the *gRNA-Mst77F* transgene was inserted into the *PBac{y+-attP-9A}VK00018* platform (53B2), using the φC31-mediated integration system [44]. *gRNA-Mst77F* flies were crossed with *nos-Cas9* males (NIG-Fly #CAS-0001). F1 virgin females with both *gRNA-Mst77F* and *nos-Cas9* alleles were collected and crossed with *y^2^cho^1^v^1^/Y; Pr Dr/TM6C, Sb Tb* males (NIG-Fly #TBX-0010). Nineteen individual F2 *v* females (both *gRNA-Mst77F* and *nos-Cas9* alleles could be simultaneously eliminated after the recombination event) were selected and established as individual lines. To screen the mutants, we performed WB using the anti-Mst77F [FL] antibody. Among the individual 19 lines, 15 lines showed no bands corresponding to Mst77F protein (data not shown). Three individual lines (*Mst77^Δ1^*, *Mst77F^Δ2^*, *Mst77F^Δ3^*) were randomly selected and the genomic DNA lesion was precisely determined by PCR amplification with the primer set 5′-ATATGGCGCCGATCTGCG-3′ and 5′-TGGTTCCTGCGGAAGTGC-3′ and sequencing.

### 4.3. Transgenic Fly generation

#### 4.3.1. P{gMst77F}

The genomic region covering the *Mst77F* gene locus (from 500-bp upstream of the transcriptional start site (TSS) to 17-bp downstream of the end of the 3′UTR) was amplified from single *w^1118^* female genomic DNA using the following primers: 5′-ATCGAATTCAGTGGTTGAAACCCCGG-3′ and 5′-ATCGCGGCCGCAATATGGGTAGAAATTTGATCAGAC-3′. The PCR fragment was cloned into the *EcoRI* and *NotI* of the *pBluescript SK+* vector (*pBS-gMst77*) and then transferred into the *pW8* vector. Transgenic flies were generated by standard P element-mediated germline transformation.

#### 4.3.2. P{mRFP1-gMst77F}

To insert the mRFP1 tag into the N-terminal of the Mst77F protein, first, the *mRFP1* coding sequence was inserted into the *pBluescript SK+* vector with *SalI* and *EcoRI* sites (*pBS-mRFP1*). PCR fragment amplified from the *pBS-gMst77F* vector with 5′-ATCGAATTCATGAGTAATCTGAAACAAAAGGA-3′ and 5′-ATCGCGGCCGCAATATGGGTAGAAATTTGATCAGAC-3′ primers was inserted into the *pBS-mRFP1* vector with *EcoRI* and *NotI* sites (*pBS-mRFP1-g2Mst77F*). Next, PCR fragment amplified from the *pBS-gMst77F* vector with 5′-ATGCGGTACCAGTGGTTGAAACCCCGG-3′ and 5′-ATCGTCGACTTTGCAACCAATTCTTGCTCG-3′ primers was inserted into the *pBS-mRFP1-g2Mst77F* vector with *KpnI* and *SalI* sites. To delete 6 bp *SalI* site before mRFP1 start codon, mutagenesis was performed using 5′-ATGGCCTCCTCCGAGGACG-3′ and 5′-TTTGCAACCAATTCTTGCTCG-3′ primers. Finally, this construct was subcloned into *pW8* with *KpnI* and *NotI* sites.

#### 4.3.3. P{gTpl94D-eGFP-6xHis}

*eGFP* cDNA without stop codon was cloned into the *pBluescript SK+* vector in *SalI* and *EcoRI* site, and sequentially 6xHis tag with stop codon was inserted in *EcoRI* and *SpeI* site. A downstream of 985 bp from just after stop codon, which included the *Tpl94D* 3′UTR was amplified from the *y w* genomic DNA with 5′-ATGACTAGTTCATCATGTCACCCACTTCAC-3′ and 5′-ATGCGGCCGCTTTCATCCAGCTGAAATCGC-3′ primers and cloned into the *pBS-eGFP-6xHis* vector in *SpeI* and *NotI* site. Next, 956 bp upstream from TSS of *Tpl94D* and open reading frame (ORF) without stop codon was amplified from the *y w* genomic DNA with 5′-ATGCGGTACCGTTATACTAAGGGCTACC-3′ and 5′-ATGCGTCGACTAAGTCTGATATGAAAATGC-3′ primers, and cloned in *KpnI* and *SalI* site. This construct was subcloned into the *pW8* vector with *KpnI* and *NotI* sites.

### 4.4. Antibody generation

#### 4.4.1. Anti-Mst77F [FL] antibody

Full-length cDNA of *Mst77F* was obtained from the Drosophila Gene Collection (DGC) (Clone ID #RH09844). *Mst77F* cDNA was amplified using a primer set (5′-CGGAATTCATGAGTAATCTGAAACAAAAGG-3′, 5′-AGCTCGAGTTACATCGAGCACTTGGGCTTG-3′), and cloned into the *EcoRI* and *XhoI* sites of the *pGEX-4T-1* plasmid (GE Healthcare), and into *EcoRI* and *SalI* sites of the *pMAL-cRI* plasmid (New England Biolabs). These plasmids were both transformed into E. coli BL21-CodonPlus (DE3)-RIL (Agilent Technologie). First, *E. coli* was incubated at 37°C for 3 h, and after adding 1 mM isopropyl beta-d-thiogalactoside (IPTG), incubated at 30°C for more 3 h. Cells were harvested, and the lysis buffer (20 mM Tris-HCL pH 8.0, 150 mM NaCl, 0.1 mM EDTA, 1% Trition X) was added, and briefly sonicated. After centrifugation, the supernatant was collected as a soluble fraction. For glutathione S-transferase (GST) fused Mst77F recombinant protein, the soluble fraction was incubated with glutathione sepharose 4B (GE Healthcare) and eluted by glutathione, and dialysed with 1× PBS buffer, and used as an antigen for the rabbit polyclonal antibody. For maltose-binding protein (MBP) fused Mst77F recombinant protein, the soluble fraction was incubated with amylose resin (New England Biolabs) and eluted by maltose, and dialysed with 1× PBS buffer, and used for the affinity purification.

#### 4.4.2. Anti-Mst35Bb antibody

Full-length cDNA of *Mst35Bb* was obtained by PCR from cDNA library of *y w* testicular total RNAs. *Mst35Bb* cDNA with *EcoRI* and *XhoI* restriction site was amplified using a primer set (5′-CGGAATTCATGAGTTCAAATAATGTAAATGAGTGC-3′, 5′-CCGCTCGAGTTACTTGCAAATCCGTCG-3′), and cloned into the *pGEX-4T-1* plasmid (GE Healthcare) in *EcoRI* and *XhoI* site, and the *pMAL-cRI* plasmid (New England Biolabs) in *EcoRI* and *SalI* site. The following steps were performed as anti-Mst77F [FL] antibody generation.

#### 4.4.3. Anti-Tpl94D antibody

The full-length cDNA of *Tpl94D* was amplified by RT-PCR from *y w* testicular total RNAs using a primer set (5′-ATGCGGATCCATGGGTAGCGTTTTAAGTAG-3′, 5′-ACTCGAGCTATAAGTCTGATATGAAAATGC-3′), and cloned into the *BamHI* and *XhoI* sites of the *pET-21a (+)* plasmid (Novagen) in *BamHI* and *XhoI* site. This plasmid was transformed into *E. coli* BL21-CodonPlus(DE3)-RIL cells (Stratagene). Inclusion body from this *E. coli* culture was suspended in 8 M urea and applied to two sequential columns, HiTrap Q and HiTrap SP (GE Healthcare) that were linked in tandem, as previously described for a recombinant histone purification [45]. The unfolded recombinant protein was dialysed and refolded. This recombinant full-length Tpl94D protein was used for both rabbit polyclonal antibody production and subsequent affinity purification.

### 4.5. Western blotting

Testicular protein extract and WB were performed as previously described [46]. Fifteen pairs of testes or seminal vesicles from 1–2 days-old males were dissected in each genotype. Mouse monoclonal anti-α-tubulin (DM1A; Sigma #T9026) was used at a 1/10 000 dilution, rabbit polyclonal anti-Mst77F [FL]; 1/5000 dilution, rabbit polyclonal anti-Mst77F [171–184]; 1/2500 dilution, rabbit polyclonal anti-Mst35Bb; 1/1000 dilution. HRP-conjugated anti-rabbit or anti-mouse (Dako #P0448 or #P0161) secondary antibodies were used at a 1/5000 dilution.

### 4.6. Fertility tests

Ten single 0–1-day-old males were crossed with two virgin *w^1118^* females each. After 7 days, parents were discarded, and the total number of adult progenies was determined at 18th day. Finally, the average number of the adult progenies in all vials was calculated.

### 4.7. Quantification of post-IC spermatid nuclear morphology

For each testis, the number of normal (bundled) and abnormal (disrupted) post-IC spermatid nuclear bundles was counted to determine the following ratio: number of normal bundles/total number of bundles. Twenty testes were analysed for each genotype and the ratio average was calculated.

### 4.8. Immunofluorescence

For each analysed genotypes, at least five pairs of testes with seminal vesicles from 0–1-day-old males were dissected, stained and observed as previously described [37]. Primary antibodies were: rabbit polyclonal anti-Mst77F [FL] (1 : 1000 dilution), rabbit polyclonal anti-Mst77F [171–184] (1 : 1000) [24], rabbit polyclonal anti-Mst35Bb (1 : 200), rabbit polyclonal anti-Tpl94D (1 : 100), mouse monoclonal anti-Histone (Millipore MABE71; 1 : 1000). Secondary antibodies were goat anti-rabbit or mouse IgG (H + L), DyLight 488 conjugated (Thermo Scientific; #35552 or #35502; 1 : 400). Phalloidin stainings (Phalloidin-FluoProbes 633A or Phalloidin-X5-FluoProbes 505; Interchim #FP-YE5230 or #FP-AZ0130; 1 : 100 dilution) were performed after secondary antibody for 30 min at room temperature. Samples were mounted in mounting medium (Dako #S3023) containing propidium Iodide (PI) (Sigma #P4170) or DRAQ5 (Cell Signalling Technology #4084 L). Confocal images were obtained using a LSM 510 confocal microscope (Zeiss). Figures were edited using ImageJ and Adobe Photoshop software.

## Supplementary Material

The Drosophila chromosomal protein Mst77F is processed to generate an essential component of mature sperm chromatin Shuhei Kimura and Benjamin Loppin. DOI 10.1098/rsob.1600207

## Supplementary Material

The Drosophila chromosomal protein Mst77F is processed to generate an essential component of mature sperm chromatin Shuhei Kimura and Benjamin Loppin. DOI 10.1098/rsob.1600207
