# Supplementary material for: The Drosophila chromosomal protein Mst77F is processed to generate an essential component of mature sperm chromatin
Source: Open Biol. 2016 Nov 3;6(11):160207. doi: 10.1098/rsob.160207 (PMC5133442; doi:10.1098/rsob.160207)

Supplemental Figures

Supplemental Figure legends

Figure S1 The *Df(3L)ri-79c* deficiency does not uncover *Mst77F*

(A) Chromatograms of genomic DNA sequences around the Cas9-gRNA cleavage site of the indicated female genotypes. The 4-bp (AAGC) deleted in the *Mst77F*$^{\Delta 1}$ allele are underlined in yellow. Black triangle: ligation point after non-homologous end-joining DNA repair on the *Mst77F*$^{\Delta 1}$ chromosome. A chromatogram corresponding to *Mst77F*$^{\Delta 1}$ was obtained for the *Mst77F*$^{\Delta 1}$/ *Df(3L)BSC452* genotype (*Df(3L)BSC452* is a deficiency that uncovers *Mst77F* with molecularly defined breakpoints [43].) In contrast, sequencing of *Mst77F*$^{\Delta 1}$/ *Df(3L)BSC562* and *Mst77F*$^{\Delta 1}$/ *Df(3L)ri-79c* DNA resulted in superimposed chromatograms, indicating the presence of both mutant and wild-type alleles. (B) Anti-Mst77F [FL] Western blotting analysis of the indicated testicular extracts. Mst77F protein is present in *Mst77F*$^{\Delta 1}$/ *Df(3L)ri-79c* extracts (lane 3). Note that Mst77F is also present in *Mst77F*$^{\Delta 1}$/ *Mst77F*^{c06969} extracts (lane 4). Anti-α-Tubulin antibody is used as loading control.

Figure S2 Histones and Tpl94D are removed at the histone-to-protamine transition in *Mst77F* mutants

(A, B) Confocal images of spermatid nuclei. Scale bars: 5 µm. (A) Anti-histone (green) signal disappears at late canoe stage in both *wild type* (upper panels) and *Mst77F* mutant (lower panels) spermatid nuclei. (B) Tpl94D-EGFP fluorescence (green) vanishes at the IC stage in both *wild type* (upper panels) and *Mst77F* mutant (lower panels) spermatid nuclei. DNA (red). F-actin (blue).

Figure S3 Deposition of Mst35Bb and Mst77F occurs independently of tNAP/Hanabi

(A-C) Confocal images of *hanabi*^{1} mutant testes. Scale bars: 10 µm. (A) General views of whole testes. Scattered spermatid nuclei in *hanabi*^{1} are stained with anti-Mst77F [FL] antibody (green). Mst77F is localized on scattered spermatid nuclei, and also in flagella (arrows). Mutant spermatid nuclei are also stained with anti-Mst77F [171-184] antibody (green) (A') and incorporate Mst77-EGFP (green)

(A''). (B) Mst35Bb is also incorporated in *hanabi[1]* mutant spermatid nuclei (anti-Mst35Bb, green). (C) Histones (anti-histones, green) are eventually removed from late *hanabi[1]* mutant spermatid nuclei, which appear slightly more compact than earlier nuclei.

Figure S4 Validation of anti-Mst77F antibodies

(A, B) Confocal images of whole testes. DNA (red). F-actin (blue). Scale bars: 10 µm. (A) In *wild type* testes (upper panel), the anti-Mst77F [FL] antibody (green) stains both spermatid nuclei (arrowheads) and flagella (arrows). In *Mst77F* mutants (middle and lower panels), the antibody faintly stains late canoe spermatid nuclei (arrowheads) before the appearance of IC. (B) The anti-Mst77F [171-184] antibody (green) decorates spermatid nuclei (arrowheads) from late canoe stage onwards (upper panels), but no specific signal is detected in *Mst77F* mutant testes (middle and lower panels).

Figure S5 Mst77F-EGFP does not rescue spermatid nuclear defects of *Mst77F* mutants

Confocal images of *P{gMst77F-EGFP}/ +; $Mst77F^{\Delta 1}$/ $Mst77F^{\Delta 2}$* spermatid nuclei at the indicated stages. Mst77F-EGFP fluorescence (green). DNA (red). F-actin (blue).

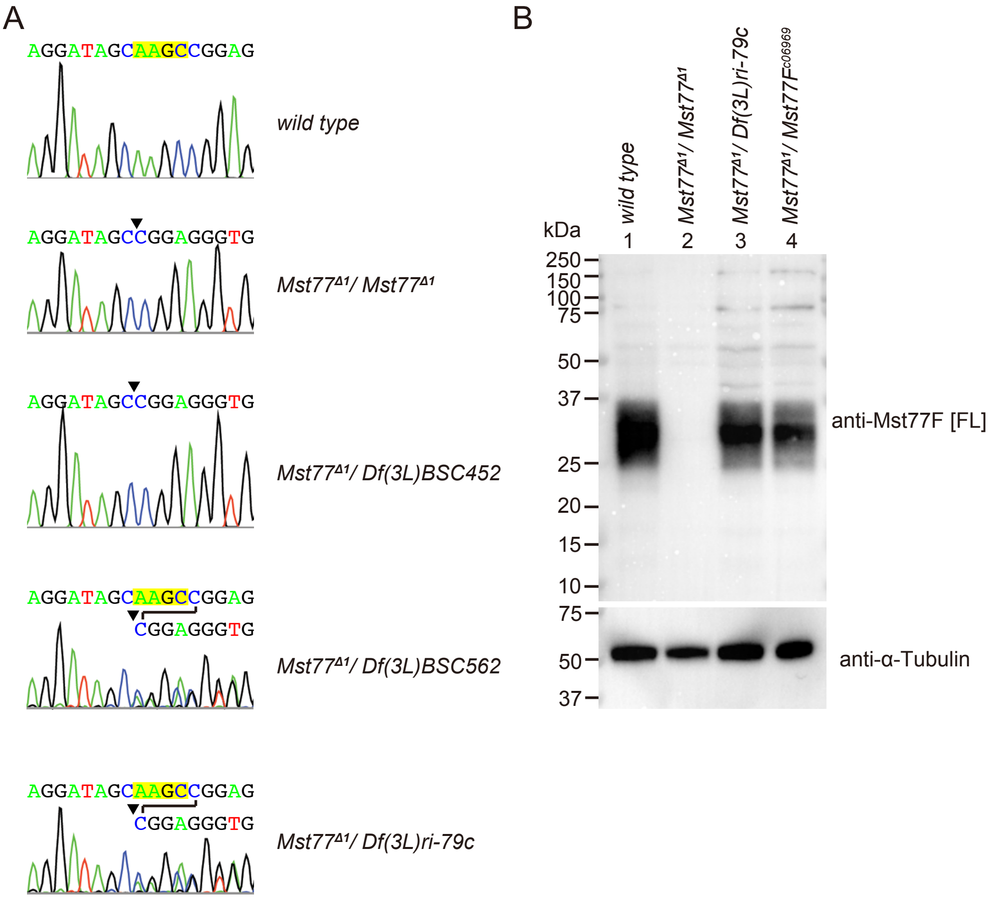
A
AGGATAGCAAGCCGGAG
wild type
AGGATAGCCGGAGGGTG
Mst77Δ1/ Mst77Δ1
AGGATAGCCGGAGGGTG
Mst77Δ1/ Df(3L)BSC452
AGGATAGCAAGCCGGAG
CGGAGGGTG
Mst77Δ1/ Df(3L)BSC562
AGGATAGCAAGCCGGAG
CGGAGGGTG
Mst77Δ1/ Df(3L)ri-79c
B
wild type
Mst77Δ1/ Mst77Δ1
Mst77Δ1/ Df(3L)ri-79c
Mst77Δ1/ Mst77Fc06969
kDa
1
2
3
4
250
150
100
75
50
37
25
20
15
10
anti-Mst77F [FL]
75
50
37
anti-α-Tubulin

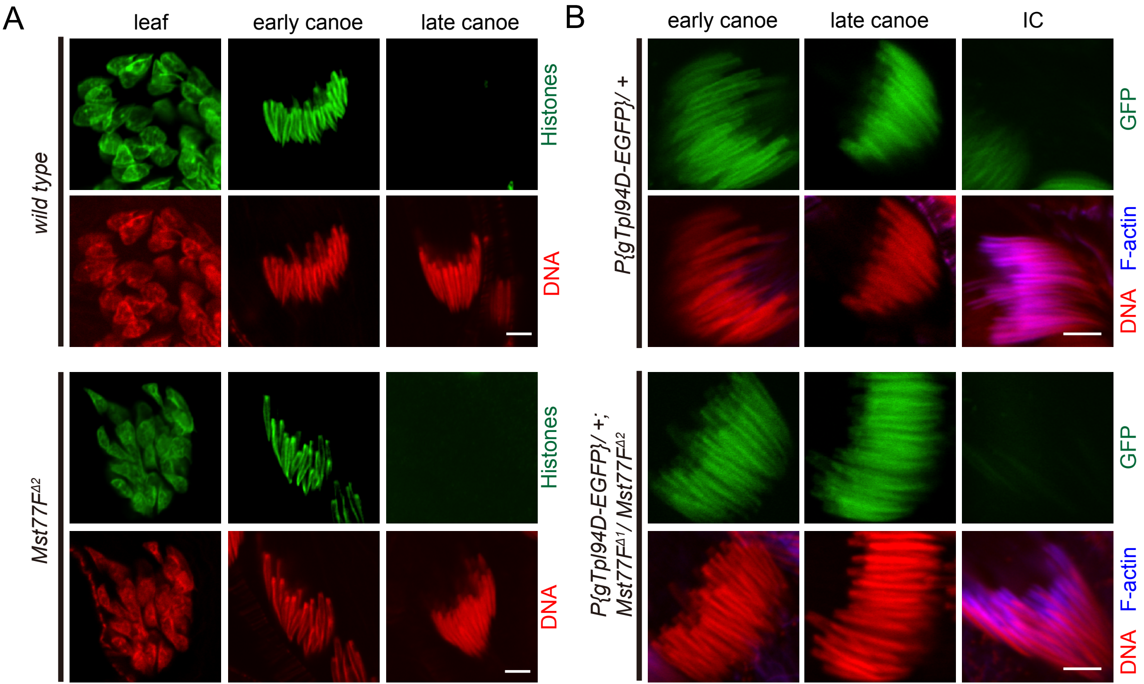
A
leaf
early canoe
late canoe
wild type
Histones
DNA
Mst77F^Δ2
Histones
DNA
B
early canoe
late canoe
IC
P{gTpl94D-EGFP}/ +
GFP
F-actin
DNA
P{gTpl94D-EGFP}/ +; Mst77F^Δ1/ Mst77F^Δ2
GFP
F-actin
DNA

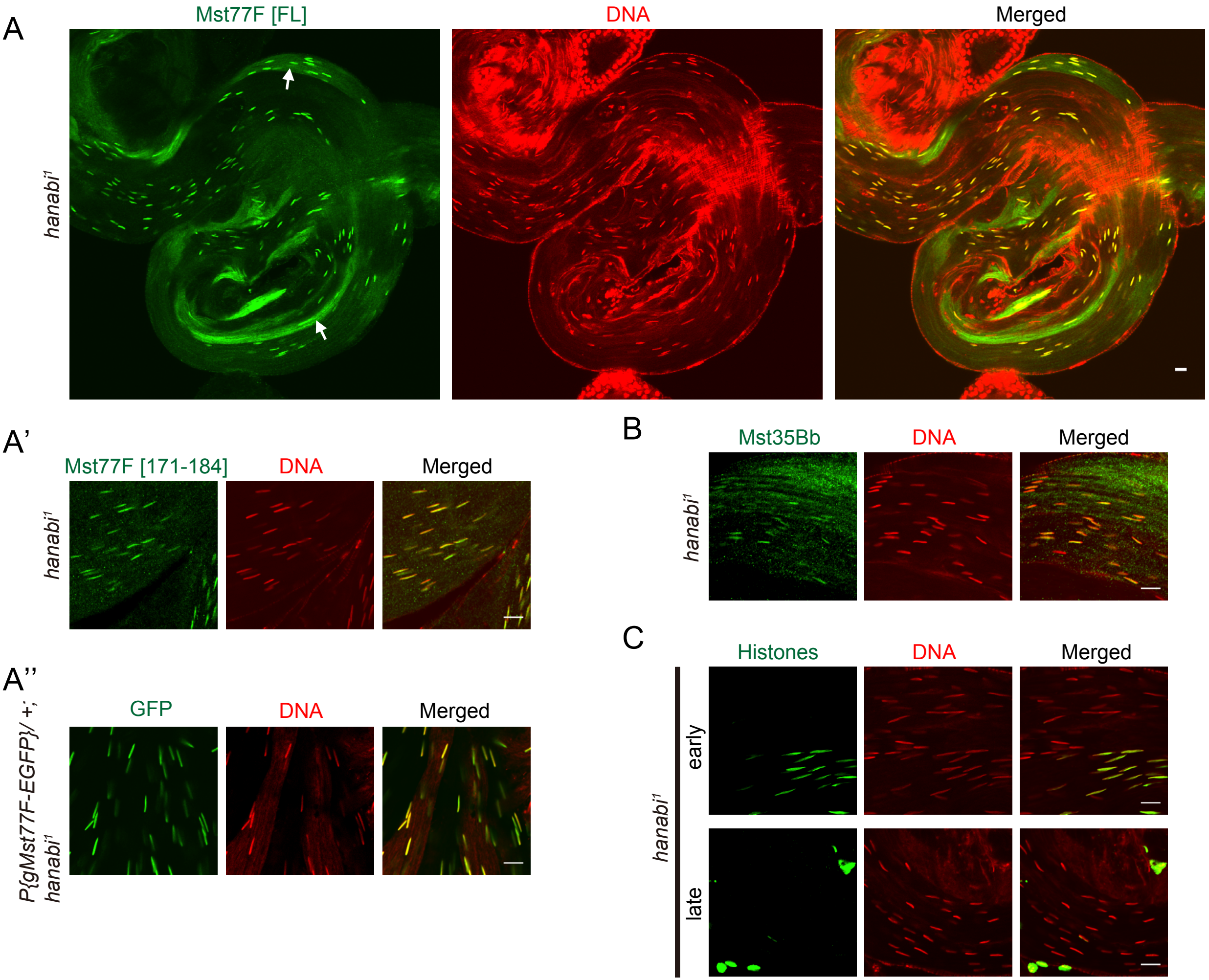
A
Mst77F [FL]
DNA
Merged
hanabi[1]
A'
Mst77F [171-184]
DNA
Merged
hanabi[1]
A''
GFP
DNA
Merged
P{gMst77F-EGFP}/ +; hanabi[1]
B
Mst35Bb
DNA
Merged
hanabi[1]
C
Histones
DNA
Merged
hanabi[1]
early
late

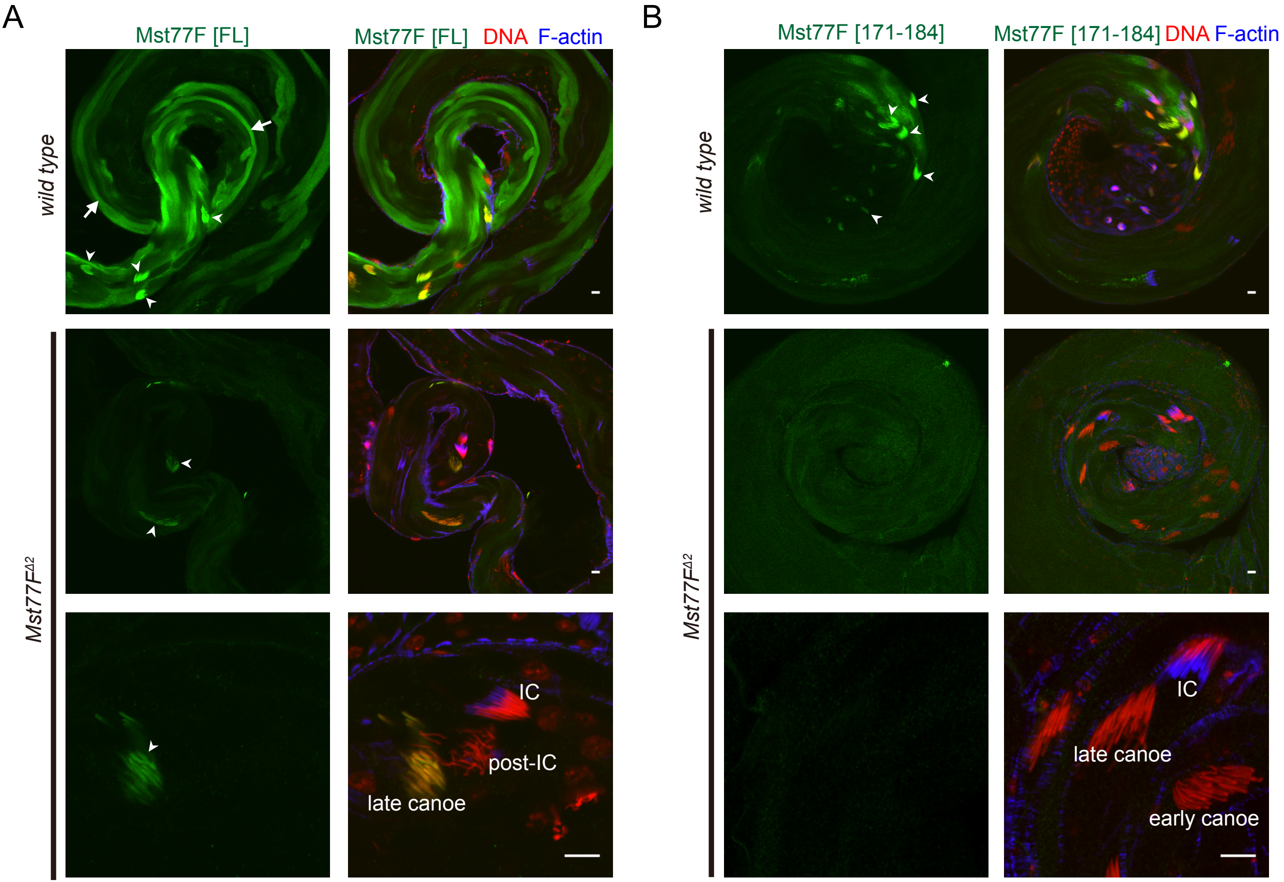
A
Mst77F [FL]
Mst77F [FL] DNA F-actin
wild type
Mst77F^Δ2
IC
post-IC
late canoe
B
Mst77F [171-184]
Mst77F [171-184] DNA F-actin
wild type
Mst77F^Δ2
IC
late canoe
early canoe

Kimura and Loppin_Fig.S5

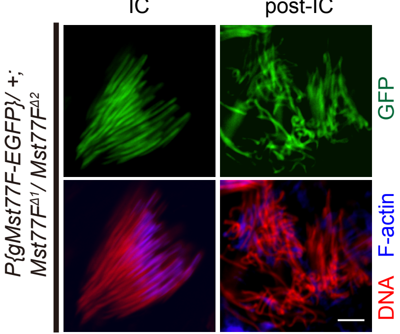

Supplement: The Drosophila chromosomal protein Mst77F is processed to generate an essential component of mature sperm chromatin Shuhei Kimura and Benjamin Loppin. DOI 10.1098/rsob.1600207 [file rsob160207supp1.pdf]
